# Epigenetic targeting of the ACE2 and NRP1 viral receptors limits SARS-CoV-2 infectivity

**DOI:** 10.1186/s13148-021-01168-5

**Published:** 2021-10-11

**Authors:** Maria Laura Saiz, Marta L. De Diego, Darío López-García, Viviana Corte-Iglesias, Aroa Baragaño Raneros, Ivan Astola, Victor Asensi, Carlos López-Larrea, Beatriz Suarez-Alvarez

**Affiliations:** 1 Translational Immunology Laboratory, Instituto de Investigación Sanitaria del Principado de Asturias (ISPA), Oviedo, Spain; 2 Department of Molecular and Cellular Biology, Centro Nacional de Biotecnología (CNB-CSIC), Madrid, Spain; 3 Intensive Care Department, Hospital Universitario Central de Asturias, Oviedo, Spain; 4 Translational Microbiology Research Group, Instituto de Investigación Sanitaria del Principado de Asturias (ISPA), Oviedo, Spain; 5 Infectious Diseases Unit, Translational Research in Infectious Diseases Group, Hospital Universitario Central de Asturias, Instituto de Investigación Sanitaria del Principado de Asturias (ISPA), Oviedo, Spain; 6 Department of Immunology, Hospital Universitario Central De Asturias, Oviedo, Spain

**Keywords:** SARS-CoV-2, ACE2, HDAC, Epigenetic, NRP1, Valproic acid

## Abstract

**Background:**

SARS-CoV-2 uses the angiotensin-converting enzyme 2 (ACE2) and neuropilin-1 (NRP1) receptors for entry into cells, and the serine protease TMPRSS2 for S protein priming. Inhibition of protease activity or the engagement with ACE2 and NRP1 receptors has been shown to be an effective strategy for blocking infectivity and viral spreading. Valproic acid (VPA; 2-propylpentanoic acid) is an epigenetic drug approved for clinical use. It produces potent antiviral and anti-inflammatory effects through its function as a histone deacetylase (HDAC) inhibitor. Here, we propose VPA as a potential candidate to tackle COVID-19, in which rapid viral spread and replication, and hyperinflammation are crucial elements.

**Results:**

We used diverse cell lines (HK-2, Huh-7, HUVEC, Caco-2, and BEAS-2B) to analyze the effect of VPA and other HDAC inhibitors on the expression of the ACE-2 and NRP-1 receptors and their ability to inhibit infectivity, viral production, and the inflammatory response. Treatment with VPA significantly reduced expression of the ACE2 and NRP1 host proteins in all cell lines through a mechanism mediated by its HDAC inhibitory activity. The effect is maintained after SARS-CoV-2 infection. Consequently, the treatment of cells with VPA before infection impairs production of SARS-CoV-2 infectious viruses, but not that of other ACE2- and NRP1-independent viruses (VSV and HCoV-229E). Moreover, the addition of VPA 1 h post-infection with SARS-CoV-2 reduces the production of infectious viruses in a dose-dependent manner without significantly modifying the genomic and subgenomic messenger RNAs (gRNA and sg mRNAs) or protein levels of N protein. The production of inflammatory cytokines (TNF-α and IL-6) induced by TNF-α and SARS-CoV-2 infection is diminished in the presence of VPA.

**Conclusions:**

Our data showed that VPA blocks three essential processes determining the severity of COVID-19. It downregulates the expression of ACE2 and NRP1, reducing the infectivity of SARS-CoV-2; it decreases viral yields, probably because it affects virus budding or virions stability; and it dampens the triggered inflammatory response. Thus, administering VPA could be considered a safe treatment for COVID-19 patients until vaccines have been rolled out across the world.

**Supplementary Information:**

The online version contains supplementary material available at 10.1186/s13148-021-01168-5.

## Background

SARS-CoV-2 is the causal agent of the coronavirus-induced disease 2019 (COVID-19) pandemic, which had cost more than 3.4 million lives worldwide by the end of May 2021 [1]. The scenario has drastically changed since the start of the pandemic. Although several vaccines have been approved for use in humans [2–6], there are many concerns regarding their worldwide accessibility, long-term efficacy, and usefulness in preventing infection with emerging virus variants. Thus, new treatments are required to manage patients with severe symptoms and those who are more vulnerable to SARS-CoV-2 infection.

SARS-CoV-2 virus enters human cells by the recognition and binding of the spike (S) protein to several cellular receptors, the angiotensin-converting enzyme 2 (ACE2) receptor being the main protein mediating viral entry [7, 8]. Initially, the receptor-binding domain (RBD) of the S1 subunit of the S protein binds the ACE2 receptor, favoring attachment to the surface of target cells [9]. Then, S protein priming by various cellular proteases facilitates the entry and fusion of the viral envelope and cellular membranes. The protease furin cleaves the S protein at the S1/S2 interface, which is further primed by the serine protease TMPRSS2. Once cleaved, the C-end rule (CendR) motif is exposed and binds to the cell surface neuropilin 1 (NRP1), which acts as another attachment factor contributing to virus entry and tropism [10, 11]. The receptors, ACE2 and NRP1, are both expressed in many human tissues, most notably the lung, but also others such as the gastrointestinal tract, kidney, and endothelial cells of the blood vessels and microvasculature. NRP1 is also found in the olfactory epithelia, neurons, and immune cells and probably contributes to the multiple systemic effects of SARS-CoV-2 [12]. Inhibition of protease activity or the engagement with ACE2 and NRP1 receptors has been shown to be an effective strategy for blocking infectivity and viral spreading. Neutralizing antibodies raised against the S protein obtained from convalescent SARS-CoV-2 patients can block viral entry through ACE2 [13]. Thus, drugs aimed at reducing or inhibiting the expression of these receptors may help prevent cellular entry of the virus and reduce its transmission to neighbor cells once the infection has taken place, thereby limiting its spread.

Some SARS-CoV-2-infected patients (20–40%) develop acute respiratory distress syndrome (ARDS) leading, in most cases, to death from COVID-19. These patients show a strong inflammatory response, similar to that of cytokine release syndrome, and increased migration of neutrophils to the lung tissue triggered by the inflammatory mediators released from local immune, epithelial, and endothelial cells, such as TNF-α and IL-6 [14]. Therefore, the blockage of viral entry and reduction of the inflammatory response are the two main targets at which most treatments are aimed. Drugs such as hydroxychloroquine and remdesivir with direct antiviral effects [15–17] and drugs with immunomodulatory properties (dexamethasone, tocilizumab, etanercept, and anakinra) [18–22] are currently used to treat COVID-19 patients.

Valproic acid (VPA; 2-propylpentanoic acid) is a branched short-chain fatty acid approved as a mood-stabilizing anticonvulsant and a broad-spectrum antiepileptic drug [23]. For more than 50 years, VPA has been used to treat epilepsy and bipolar disorders, and as migraine prophylaxis. More recent discoveries about VPA’s function as an inhibitor of histone deacetylases (HDACs) have aroused new interest in this epigenetic inhibitor as a cancer treatment [24, 25]. Moreover, due to its low cost, favorable side effect profile, and the ease with which it crosses the blood brain barrier, VPA is an attractive candidate drug for a variety of possible indications. However, it is contraindicated in pregnancy due to the high risk of congenital malformations [26].

To date, several in vitro and animal studies have demonstrated the potent anti-inflammatory and antiviral effects of VPA, two of the key events in targeting severe COVID-19. As an HDAC inhibitor, VPA decreases the production of NF-kB-induced proinflammatory cytokines such as TNF-α and IL-6, promotes the differentiation to Th2 and regulatory T cells, and modulates neutrophil migration and IFN-γ-activated macrophage response, among other activities [27–30]. Additionally, VPA has demonstrated direct antiviral activity against enveloped viruses such as vesicular stomatitis virus (VSV), Semliki forest virus (SFV), West Nile virus (WNV), vaccinia virus (VACV), and lymphocytic choriomeningitis virus (LCMV), by inducing the generation of highly unstable viral particles through its properties as a disruptor of lipid composition of cellular membranes [31, 32]. More recently, the antiviral efficacy of VPA against herpes simplex virus 1 (HSV-1) has been reported, showing that patients receiving VPA treatment had a lower risk of herpesvirus infection than those not treated with this compound [33].

Given the aforementioned effects, some letters and reviews have already championed the therapeutic potential of VPA for COVID-19 patients [34–36]. Nevertheless, so far, the actual mechanisms by which VPA could be useful in SARS-CoV-2 infection have not been addressed. Our results demonstrate that VPA not only reduces the expression of the S protein receptors, ACE2 and NRP1, but also blocks viral production, restricting viral spread between neighboring cells. Also, VPA acts as a potent immunosuppressor, reducing the production of proinflammatory cytokines. All these effects make VPA a suitable candidate for treating COVID-19 patients.

## Results

### VPA reduces ACE2 and NRP1 expression in cells from different tissues by an HDAC-dependent mechanism

Recent studies have yielded powerful evidence that angiotensin-converting enzyme 2 (ACE2) is the major cellular-entry receptor for the SARS-CoV-2 virus, and ACE2 expression may increase tissue susceptibility to SARS-CoV-2 infection [37]. In fact, the different ACE2 levels in a variety of tissues and cell types indicate the specific vulnerability of each organ to the infection [38].

**Fig. 1:**
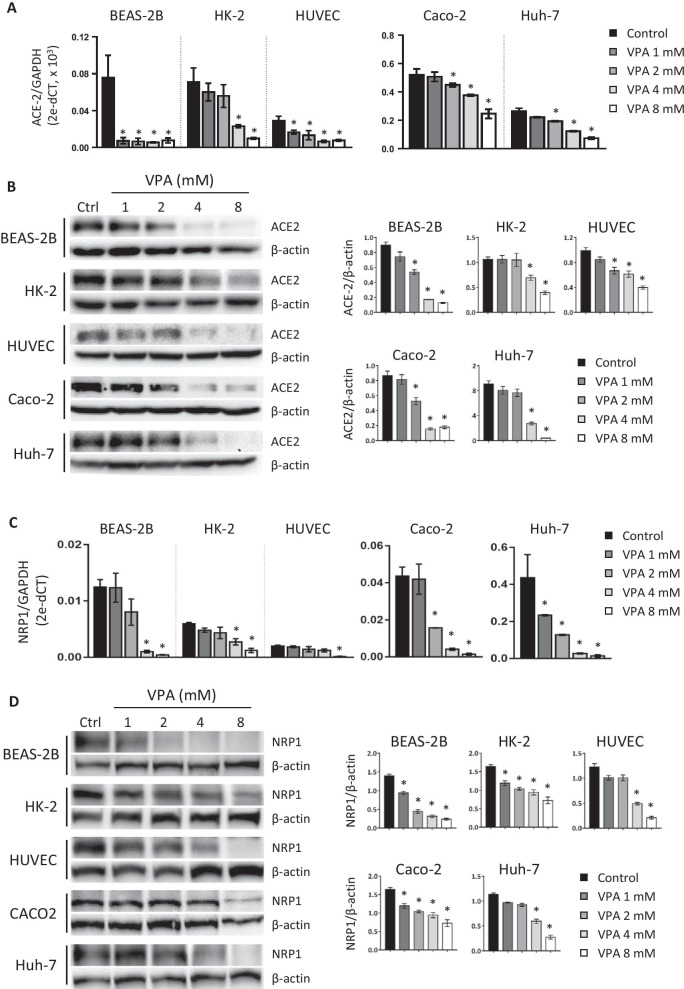
VPA downregulates ACE2 and NRP1 expression in cell lines from different sources. Human cell lines (BEAS-2B, HK-2, HUVEC, Caco-2, and Huh-7) were treated with different doses of VPA (1, 2, 4, and 8 mM) for 24 h. VPA was diluted in culture medium, which was also used as control. Expression of the ACE2 (**a**, **b**) and NRP1 (**c**, **d**) receptors was assayed by RT-qPCR (**a**, **c**) and western blot (WB) (**b**, **d**). *GADPH* and β-actin genes were used as endogenous controls to quantify mRNA and protein levels, respectively. Transcription levels were calculated by the 2^−ΔCT^ method (ΔCT: CT gene test—CT endogenous control). Data are presented as the mean ± SD of at least three independent experiments. *p < 0.05

Here, we analyzed the effect of VPA on ACE2 expression in human cell lines of different origins (lung, kidney, primary endothelium, for BEAS-2B, HK-2, and HUVEC cells, respectively) and in two tumor-derived cell lines (hepatocarcinoma and colorectal carcinoma, for Huh-7 and Caco-2 cells, respectively). To this end, we treated cells for 24 h with a range of nontoxic VPA concentrations (1–8 mM), previously determined by Annexin V/7AAD assay (Additional file 1: Fig. S1) and quantified the transcriptional and protein levels of ACE2 expression. First, we confirmed that all untreated cells show a high level of expression of ACE2 (Fig. 1A, B). Treatment with VPA significantly decreased ACE2 expression at the mRNA (Fig. 1A) and protein (Fig. 1B) levels in a dose-dependent manner in all the analyzed cell lines. Furthermore, protein levels of ACE2 were almost fully inhibited at a dose of 8 mM (Fig. 1A, B). We further evaluated the effect of VPA on the expression of NRP1, an additional host factor that facilitates SARS-CoV-2 entry. Interestingly, similar results to those shown for ACE2 were observed. VPA drastically reduced the mRNA expression levels of NRP1 in a dose-dependent fashion, which was also correlated with a decrease at the protein level (Fig. 1C, D). However, the transcriptional levels of other genes such as *CTSL1* (endosomal cysteine protease Cathepsin L, used for SARS-CoV for S protein priming), *DPP4 *(the primary receptor for MERS-CoV), and *RFX5* (transcription factor essential for MHC class II expression) in HK-2 and Huh-7 cells remain unchanged after VPA treatment (Additional file 2: Fig. S2) [13, 39, 40]. These results therefore indicate that VPA specifically interferes with the transcriptional regulation of ACE2 and NRP1 receptors, impairing their expression, independently of cell type or whether the cell lines are derived from tumors.

**Fig. 2:**
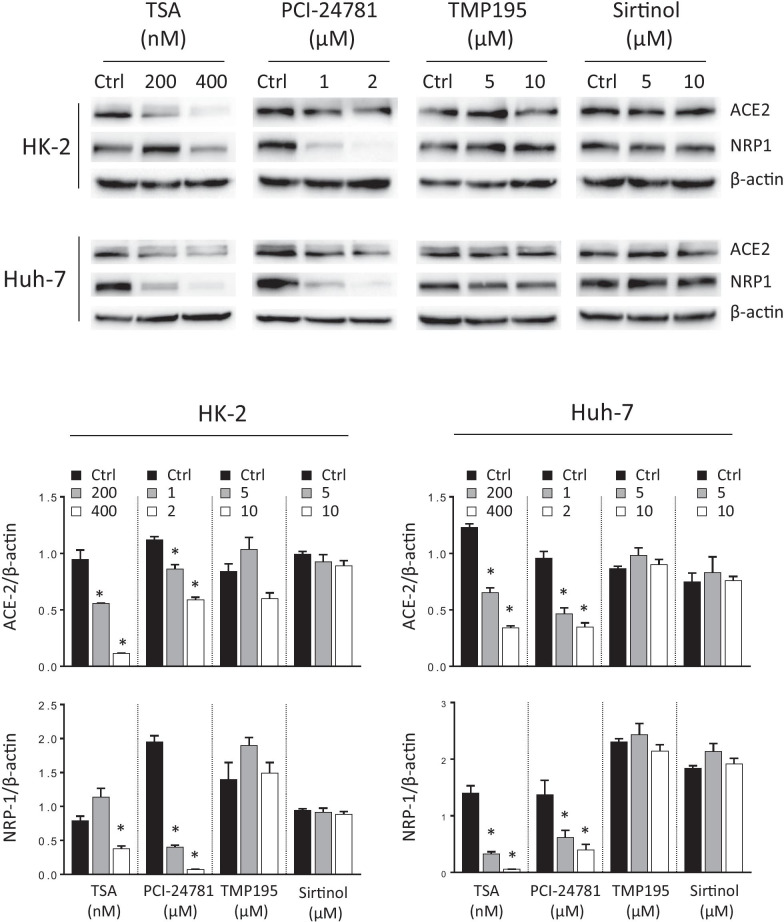
Reduced expression of ACE2 and NRP1 is mediated by blocking histone deacetylases. HK-2 and Huh-7 cell lines were treated with different histone deacetylase (HDAC) inhibitors; trichostatin A (TSA, specific for class I and II HDACs, 200 and 400 nM), PCI-24781 (specific for class I and IIb HDACs, 1 and 2 µM), TMP-195 (specific for class IIa HDACs, 5 and 10 µM) and sirtinol (specific for SIRT1 and SIRT2, a class III HDAC, 5 and 10 µM) for 24 h. All inhibitors were prepared in DMSO, which was also used as control. Expression of the ACE2 and NRP1 receptors was assayed by WB using β-actin as endogenous control. Data are presented as the mean ± SD of at least three independent experiments. *p < 0.05

One of the most important characteristics of VPA is its ability to inhibit histone deacetylases (HDACs), thereby increasing the acetylation levels in lysine residues from histones and nonhistone proteins [41, 42]. VPA is known as a pan-inhibitor, since it inhibits class I (HDAC 1–3, and 8) and IIa (HDAC 4, 5, 7, and 9), but not those of other HDAC classes [43]. To address whether the effect of VPA on those receptors was mediated by this mechanism, we assayed the effect of other HDAC inhibitors on the ACE2 and NRP1 expression in HK-2 and Huh-7 cell lines. Trichostatin A (TSA) is a reversible class I and II mammalian HDAC inhibitor, PCI-24781 specifically inhibits class I and IIb HDACs, TMP-195 is specific for class IIa HDACs and sirtinol was used as a specific SIRT1 and SIRT2 (class III HDACs) inhibitor. Data show that TSA and PI-24781 lowered ACE2 and NRP1 mRNA and protein levels, while their levels were unchanged by TMP-195 and sirtinol treatments (Fig. 2 and Additional file 3: S3). These results validate the hypothesis that HDAC inhibition is the main mechanism involved in the decrease of ACE2 and NRP1 expression, likely due to the blockage of the class I HDACs that are inhibited by VPA, TSA, and PCI-24781.

### Pretreatment with VPA inhibits SARS-CoV-2 infection

**Fig. 3:**
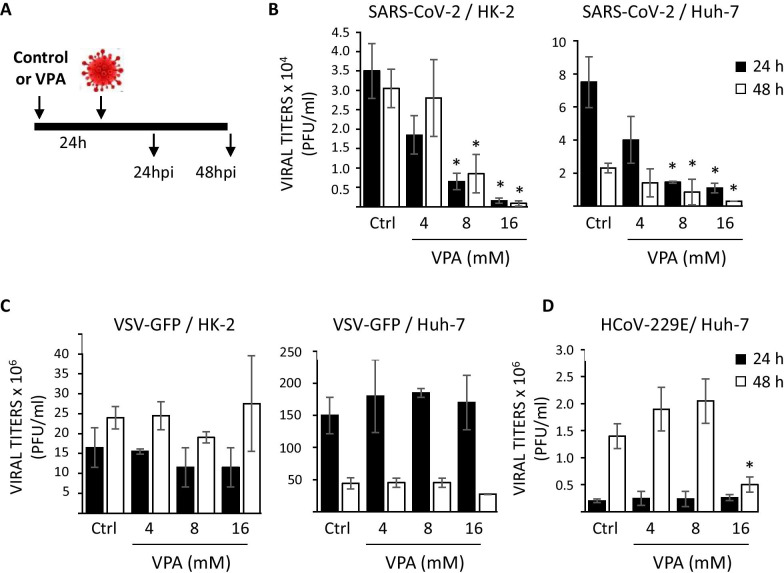
Pretreatment with VPA reduces the viral infection mediated by SARS-CoV-2. **a** Schematic of the experiment to determine the prophylactic effect of VPA on viral production. HK-2 and Huh-7 cells were treated with VPA at 4, 8, and 16 mM concentrations, or left untreated (control cells). At 24 h after treatment, culture medium was replaced by fresh medium without VPA. Cells were infected with the different viruses independently and the supernatants were collected at 24 and 48 h post-infection (hpi) for virus titration by plaque assay. Viruses analyzed were SARS-CoV-2 (MOI 0.5, **b**), VSV-GFP (MOI 0.01, **c**), and HCoV-229E (MOI 0.1, **d**). The latter was assayed only in Huh-7 cells. Virus titers were determined by a lysis plaque assay and represented in absolute numbers as plaque-forming units per ml (PFU/ml). Data are represented as the mean ± SD of three independent experiments. *p < 0.05

As VPA reduces the expression of ACE2 and NRP1, we analyzed whether the pretreatment of cells with VPA could affect SARS-CoV-2 infection. To this end, we used cell lines susceptible to SARS-CoV-2 infection, such as the tumor-derived Huh-7 cell line and the non-tumoral HK-2 cell line. Samples of Huh-7 and HK-2 cells were pretreated for 24 h with different concentrations of VPA (4–16 mM). After that, culture medium was replaced by fresh medium without VPA, and cells were then infected and virus titers analyzed 24 and 48 hpi (Fig. 3A). Previously, we evaluated the effect of VPA on cell proliferation of HK-2 and Huh-7 cells lines by MTT assay. It is of note that even at the highest concentration of VPA (16 mM), the inhibition of the proliferation was never greater than of 50% (Additional file 4: Fig. S4).

The results show that at 24 hpi, virus titers were more than 95% lower (22-fold difference, measured in PFU/ml) in HK-2 and more than 85% lower (sevenfold difference, measured in PFU/ml) in Huh-7 cells treated with the highest concentration of VPA (16 mM) compared with control cells (Fig. 3B and Additional file 5: Fig. S5). Similarly, at 48 hpi, virus titers in cells treated with the highest concentration of VPA were reduced by more than 97% and 85% (35- and eightfold decrease in HK-2 and Huh-7 cells, respectively, measured in PFU/ml) (Fig. 3B and Additional file 5: Fig. S5). Moreover, virus titers at 24 hpi, in HK-2 and Huh-7 cells pretreated with VPA at the highest concentration, compared with virus titers measured just after virus absorption (0 hpi), were only increased 2- and 15-fold, respectively, compared with the 50-and 100-fold increases in control cells, respectively. These results indicate that virus production was very limited, particularly in HK-2-treated cells (Additional file 5: Fig. S5). There was also a dose-dependent effect on the reduction of virus production (Fig. 3B and Additional file 5: Fig. S5), strongly suggesting that the pretreatment of cells with VPA reduces SARS-CoV-2 production. This effect is independent of the tumoral or non-tumoral origin of the cells.

To determine whether this effect also applies to other enveloped viruses, HK-2 and Huh-7 cells were infected with VSV-GFP and with another human coronavirus, the HCoV-229E (Fig. 3C, D). In contrast to VPA-treated SARS-CoV-2-infected cells, in which we observed a significant decrease in the yields of infectious viruses, no significant effect on the virus yields was detected in HK-2 and Huh-7 cells infected with VSV-GFP and pretreated with VPA, even at the highest concentration, compared with the control cells (Fig. 3C, D). Moreover, regarding HCoV-229E infection experiments, only small (2.5-fold, measured in PFU/ml) differences were detected with the highest VPA concentration (16 mM) and only at 48 hpi (Fig. 3C, D) in Huh-7 cells. HK-2 cells were not susceptible to HCoV-229E infection (data not shown). Thus, these data show that pretreatment with VPA significantly reduced infection with SARS-CoV-2 but not with other viruses. The null or negligible effect observed with VSV-GFP and HCoV-229E, whose entry into mammalian cells is independent of those receptors, suggests that the outcome could be mediated, at least in part, by the effect of VPA on ACE2 and NRP1 expression.

**Fig. 4:**
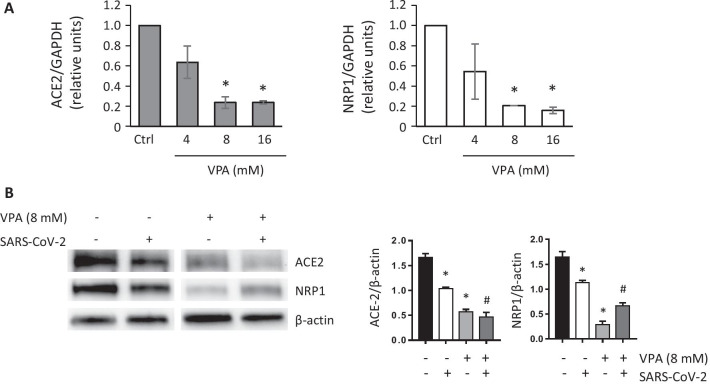
VPA reduces ACE2 and NRP1 expression after SARS-CoV-2 infection. **a** Huh-7 cells were treated for 24 h with VPA at 4, 8, and 16 mM, or left untreated (control cells). They were then infected with SARS-CoV-2 (MOI 0.5) in the absence of VPA. The expression of ACE2 and NRP1 was analyzed by RT-qPCR at 24 hpi. GAPDH was used as the endogenous control and transcription levels were calculated by the 2^−ΔCT^ method. The results are represented as levels relative to untreated (control) cells. **b** Huh-7 cells were treated with VPA (8 mM) for 24 h and further infected with SARS-CoV-2 (MOI 0.5) in the absence of VPA for additional 24 h. The levels of ACE2 and NRP1 proteins were assayed and quantified by WB, using β-actin as endogenous controls. All samples were analyzed at the same time and came from the same blot. Data are represented as the mean ± SD of two independent experiments. *p < 0.05, compared with control; ^#^p < 0.05, compared with SARS-CoV-2- infected cells

To obtain further evidence to support this hypothesis, samples of Huh-7 cells were pretreated for 24 h with different concentrations of VPA (4, 8, and 16 mM) and then infected with SARS-CoV-2 for 24 h. At the end of the experiment, ACE2 and NRP1 expression levels were quantified by RT-PCR and western blot. We observed that cells pretreated with VPA and additionally infected with SARS-CoV-2 maintained low levels of transcription for ACE2 and NRP1 compared with untreated control cells in a dose-dependent manner (Fig. 4A). We also confirmed that, although SARS-CoV-2 infection slightly downregulates ACE2 expression, as previously suggested [44], the effect of VPA (8 mM) on ACE2 and NRP1 protein levels was stronger and persisted at almost negligible levels 24 hpi (Fig. 4B). Thus, our findings could explain the potential effect of the pretreatment with VPA on the damaged infectivity of SARS-CoV-2.

### Treatment with VPA post-infection reduces viral replication without affecting the synthesis of viral N protein

**Fig. 5:**
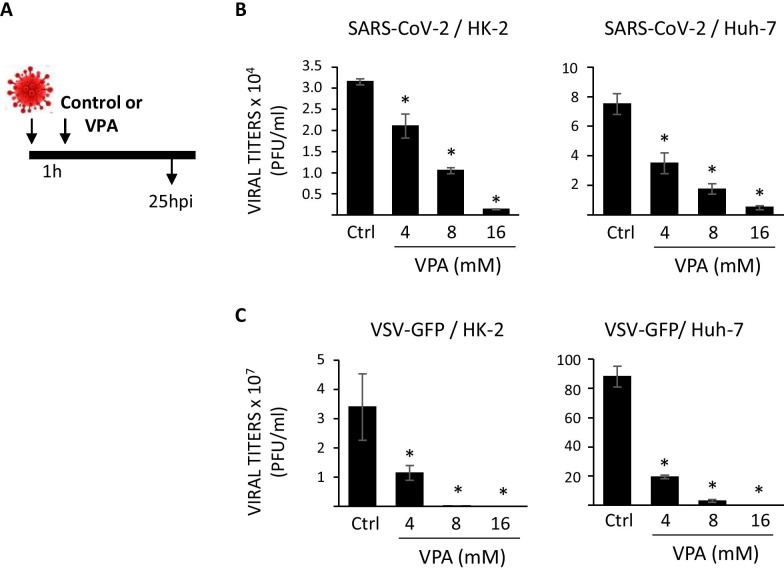
Therapeutic effect of VPA on SARS-CoV-2 and VSV-GFP yields. **a** Schematic of the experiment to determine the therapeutic effect of VPA on viral yield. HK-2 and Huh-7 cells were infected with SARS-CoV-2 (MOI of 0.5) or VSV-GFP (MOI of 0.01) and 1 hpi, the cells were treated with VPA at 4, 8, and 16 mM, or left untreated (control cells) for an additional 24 h. Virus titers of SARS-CoV-2 (**b**) and VSV-GFP (**c**) were determined by a lysis plaque assay and are represented in absolute numbers as plaque-forming units per ml (PFU/ml). Data are represented as the mean ± SD of triplicate experiments. *p < 0.05

We wanted to evaluate the effect of VPA once SARS-CoV-2 infection has been established. To this end, HK-2 and Huh-7 cells were infected with SARS-CoV-2 or VSV-GFP and treated 1 hpi with different concentrations (4–16 mM) of VPA. SARS-CoV-2 and VSV-GFP viral titers were measured at 24 hpi (Fig. 5A). The results showed that SARS-CoV-2 titers were more than 95% and 93% lower (23- and 15-fold, measured in PFU/ml) in HK-2 and Huh-7 cells, respectively, when the cells were treated with the highest VPA concentration compared with control cells (Fig. 5B and Additional file 6: Fig. S6). In this case, similar results were observed with the VSV-GFP virus, as VPA treatment after infection decreased titers by more than 99% (4,000,000- and 235,000-fold, measured in PFU/ml) in both cell lines at the highest VPA concentration, relative to control cells (Fig. 5C and Additional file 6: Fig. S6). Moreover, a dose-dependent effect on the reduction of infectious virus yields was observed under all conditions, and this effect applied both to tumoral- and non-tumoral-derived cell lines.

These findings suggested that VPA might inhibit postentry steps of the life cycles of these viruses, reducing the quantity of infectious viruses. To rule out the possibility that the effect of VPA could be due to its ability to reduce cell growth, proliferation assays in HK-2 and Huh-7 cell lines were carried out in the presence of different concentrations of VPA at 24 h. The results showed that VPA reduced cell proliferation but never reached > 50% inhibition, even at the highest dose (Additional file 7: Fig. S7). Thus, these results suggest that the reduction in the viral titers induced by VPA is independent of its anti-proliferative effect.

**Fig. 6:**
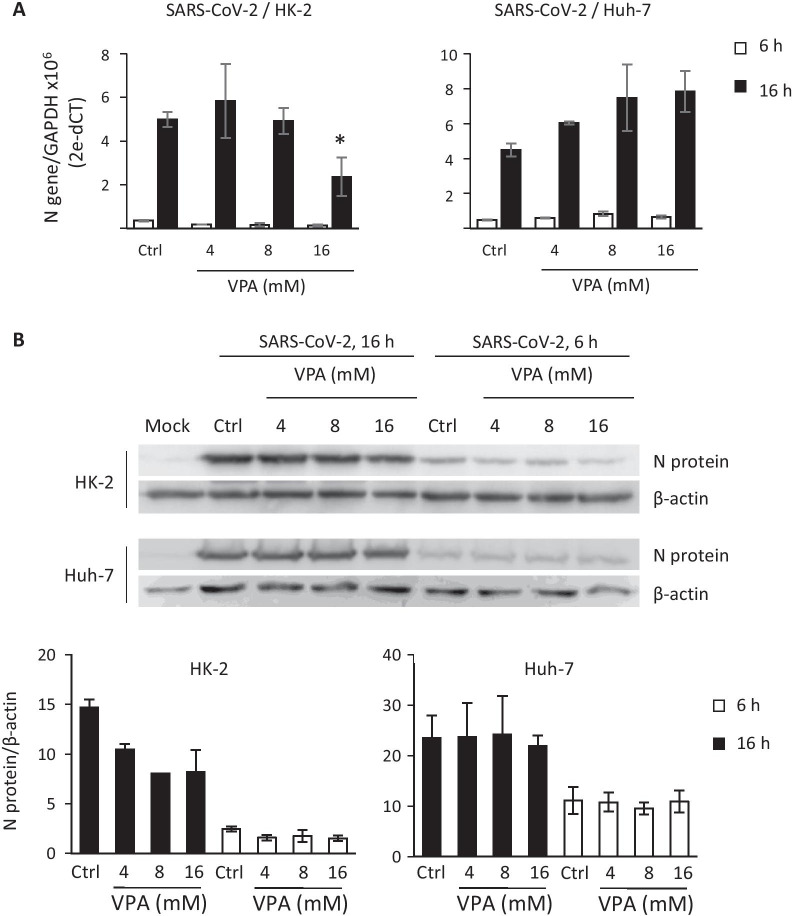
VPA does not interfere with the synthesis of SARS-CoV-2 proteins. HK-2 and Huh-7 cells were infected with SARS-CoV-2 (MOI 0.5), and treated with VPA (4, 8, and 16 mM) 1 hpi. gRNA/sg mRNA expression levels (**a**) and protein levels (**b**) of protein N were analyzed at 6 and 16 hpi. *GADPH* (**a**) gene and β-actin (**b**) were used as endogenous controls for quantification of N gRNA/sg mRNA and protein levels. Data are presented as the mean ± SD of at least three independent experiments. Mock; mock-infected cells (**b**). * p < 0.05

To analyze whether VPA inhibits SARS-CoV-2 replication and transcription, HK-2 and Huh-7 cells were infected with SARS-CoV-2 for 1 h, then treated with VPA (4–16 mM). Total RNAs were extracted at 6 and 16 hpi, and the levels of genomic (g) (gRNA) and subgenomic (sg) mRNAs (sg mRNAs) for the *N* gene were evaluated by RT-qPCR. No statistically significant differences were detected in control cells compared with VPA-treated cells, except for HK-2 cells, in which a slight decrease was observed at the highest VPA concentration (Fig. 6A). Likewise, when the N protein levels were detected in infected cells by western blot analysis, no difference was observed between the control-infected cells and VPA-treated infected cells of the Huh-7 cell line, and there was only a negligible reduction with the 16 mM VPA dose in HK-2 cells (Fig. 6B). Taken together, these findings suggest that VPA inhibits the viral yield without interfering with the transcription and translation of viral proteins.

### VPA reduces the inflammatory response triggered by TNF-α induction and virus infection

It has been extensively shown that SARS-CoV-2-induced pathology is due, at least in part, to an exacerbated inflammatory response [14, 45–47]. In addition, several studies have described how VPA can block the expression of inflammatory cytokines, including TNF-α and IL-6 [48, 49].

**Fig. 7:**
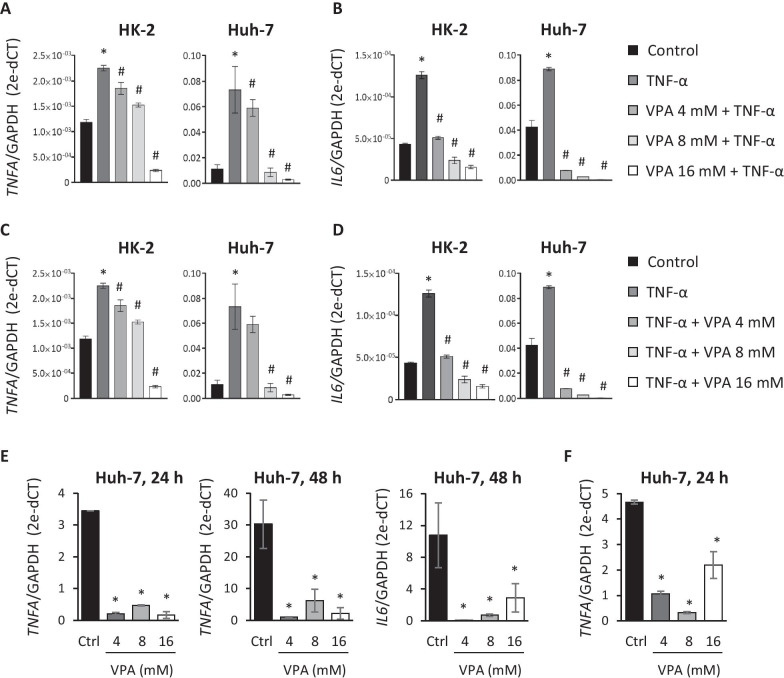
Treatment with VPA inhibits the inflammatory response triggered by TNF-α induction and SARS-CoV-2 infection. **a**,** b** HK-2 and Huh-7 cells were treated with VPA (4, 8, and 16 mM) for 24 h and (10 ng/ml) was added in the final 3 h. **c, d** HK-2 and Huh-7 cells were cultured with TNF-α (10 ng/ml) for 3 h and without removing the culture medium, and VPA (4, 8, and 16 mM) was added for an additional 24 h. TNF-α and IL-6 expression was quantified by RT-qPCR and represented as n-fold induction over the levels of mock-treated cells. **e** Huh-7 cells were treated with VPA (4, 8, and 16 mM) for 24 h before SARS-CoV-2 infection (MOI 0.5), and TNF-α and IL-6 expression was evaluated by RT-qPCR at 24 and/or 48 hpi and represented as the n-fold induction over the levels of mock-infected cells. **f** Huh-7 cells were infected with SARS-CoV-2 and 1 hpi, cells were left untreated (control) or treated with 4, 8, and 16 mM of VPA for 24 h. TNF-α expression was analyzed by RT-qPCR and represented as the n-fold induction over the levels of mock-infected cells. All samples were normalized relative to GAPDH expression using the 2^−ΔCT^ method. Data are represented as the mean ± SD of at least three independent experiments. *p < 0.05

In accordance with this, we initially evaluated the effect of VPA in the expression of the pro-inflammatory cytokines, IL-6 and TNF-α, in HK-2 and Huh-7 cells, when the VPA was added before or after TNF-α induction. First, we checked that treatment with TNF-α (10 ng/ml) increased the expression of both cytokines under all the conditions analyzed (Fig. 7A–D). To measure the preventive effect of VPA, we cultured HK-2 and Huh-7 cells with different doses of VPA (4, 8, and 16 mM) for 24 h, adding TNF-α 3 h before the end of the culture period. Expression levels of TNF-α and IL-6 were quantified by RT-qPCR. The results showed that VPA limited the increased expression of TNF-α and IL-6 in both cell lines in a dose-dependent way (Fig. 7A, B. The VPA effect was mainly observed in IL-6 expression, where the lowest dose of VPA assayed (4 mM) reduced its expression to levels seen in controls, at most (Fig. 7B). We also determined whether VPA had the same anti-inflammatory effect when it was added after TNF-α induction. Cell lines were treated with TNF-α for 3 h and, without removing the culture medium, VPA was added for an additional 24 h. We again observed that the TNF-α and IL-6 mRNA levels were upregulated by TNF-α, although the increase was counteracted by the presence of VPA (Fig. 7C, D).

To establish whether VPA treatment has similar effects after SARS-CoV-2 infection, Huh-7 and HK-2 cells were treated with VPA for 24 h before infection. Cells were then infected and the expression levels of TNF-α and IL-6 were evaluated by RT-qPCR at 24 and 48 hpi. The results showed that TNF-α expression levels were induced in control Huh-7 cells after 24 hpi (± threefold) but not in infected cells pretreated with VPA (Fig. 7E). These results were more evident at 48 hpi, at which time TNF-α expression is around 30 times higher in SARS-CoV-2-infected Huh-7 cells, whereas in VPA-pretreated cells its expression had only increased 2- to fivefold (Fig. 7E). Similar results were observed in IL-6 expression, although in that case the induction was only detected at 48 hpi (Fig. 7E). Surprisingly, after SARS-CoV-2 infection, we could not detect the induction of TNF-α or IL-6 in HK-2 cells, even after 48 hpi (data not shown). In summary, VPA treatment could also prevent the inflammatory response triggered as a consequence of SARS-CoV-2 infection. In order to determine whether VPA could have a therapeutic effect and reduce the expression of pro-inflammatory cytokines when administered after SARS-CoV-2 infection, Huh-7 cells were infected and VPA was added 1 hpi for an additional 24 h. Once again, a decrease in TNF-α expression was observed for VPA-treated SARS-CoV-2-infected cells compared with untreated, infected cells (Fig. 7F). As reported above, IL-6 expression was not evaluated because the level of that cytokine had not increased 24 hpi.

Overall, these findings indicate that besides reducing the extent of production of infectious viruses, VPA also suppresses the expression of inflammatory cytokines after infection. This effect is probably not linked to the effect of VPA on limiting virus production since the same effect is observed by inducing the expression of inflammatory cytokines by TNF-α, in the absence of virus infection. This gives VPA an added value for controlling the exacerbated inflammatory response upon SARS-CoV-2 infection, which can lead to acute respiratory distress syndrome, and a worse prognosis for COVID-19.

## Discussion

Infection by SARS-CoV-2 may cause patients to develop an exacerbated inflammatory response and extensive spread of the virus to multiple organs, leading to severe complications such as pneumonia and multiorgan dysfunction [50]. Here we show that VPA, a drug approved for the treatment of epilepsy, bipolar disorders, and migraines, can reduce the expression of the main cellular receptors of SARS-CoV-2, ACE2 and NRP1, in cell lines from a variety of human sources and thereby impair the spread of the virus to neighboring cells. Moreover, in SARS-CoV-2-infected cells, treatment with VPA reduces the production of infectious viruses without significantly modifying viral RNA and protein synthesis and, according to one of the most widespread effects described for VPA, this drug can also inhibit the production of the TNF-α and IL-6 proinflammatory cytokines triggered by SARS-CoV-2 infection.

Our results establish that ACE2 and NRP1 expression is regulated by epigenetic mechanisms. VPA specifically reduces the transcriptional levels of both SARS-CoV-2 receptors, an effect that is probably mediated by its activity as an HDAC inhibitor. Teodori et al*.* carried out a bioinformatic analysis to show that the HDAC pathway is the key to the pathogenicity of SARS-CoV-2 [51]. They proposed that activation of AT1R (angiotensin II receptor type 1) by the excessive accumulation of angiotensin II (AngII) leads to the stimulation of HDACs that upregulate ACE2 expression. Moreover, the clinically used HDAC inhibitor, panobinostat, suppresses the expression of ACE2 in the gastric cancer cell line, KATOIII [52]. Given that observation and our own findings, the blockage of HDAC activity by VPA or other specific inhibitors reduces ACE2 expression. Additionally, the VPA-induced ACE2 downregulation was maintained even during SARS-CoV-2 infection. HDACs are normally associated with transcriptional repression, so their inhibition is usually associated with increased gene expression. However, transcriptional repression arising from inhibition of HDACs is much less well known. Recent reports have shown that HDACs could also positively regulate transcription by stimulating transcription elongation using mechanisms involving enhancer RNA synthesis and displacement of negative elongation factor (NELF) at promoters [53]. Other studies have demonstrated that HDAC inhibitors can repress gene transcription by blocking RNA polymerase II elongation in breast cancer, which preferentially acts on highly expressed genes as well as on high copy number genes [54]. Thus, the action of VPA on ACE2 expression could be mediated through the aforementioned mechanisms, by an indirect mechanism in which this inhibitor might modify the acetylation levels in the regulatory region of genes, or directly by acting on transcription factors that are involved in the transcriptional repression of ACE2. Whatever the explanation, these epigenetic-regulated mediators need to be identified.

An earlier study had shown that SIRT1, a class III HDAC, binds to the promoter region of ACE2, reducing its expression under cell stress [55]. The angiotensin II/AT1R axis plays a key role in the aging of the kidney increasing chronic inflammation and cell senescence. However, the angiotensin 1–7 (Ang 1–7)/Mas receptor (MasR) axis, mediated by the activation of ACE2, counterbalances that effect. Administration of the sirtuin inhibitor, resveratrol, to aged mice favors the enhancement of the Ang 1–7/MasR axis mediated by ACE2 exerting a protective effect [56]. By contrast, we observed that the growth of several cell lines under physiological conditions and in the presence of sirtinol (a specific SIRT1 and SIRT2 inhibitor) does not modify ACE2 expression. Therefore, the regulation mediated by sirtuins could be restricted by the cellular state, since sirtuins are known to be involved in stress responses [57, 58]. A recent study that aimed to evaluate the inflammatory response induced by SARS-CoV-2 in the heart found that BRD4, a member of the BET family of bromodomains, is responsible for triggering cardiac injury and dysfunction [59]. Blockage of BRD4 action with epigenetic treatments reduces ACE2 expression and viral titers and protects against cardiac dysfunction in the K18-hACE2 mouse model.

The mechanisms of epigenetic regulation involved in NRP1 transcription are less well known, but butyrate, an HDAC inhibitor derived from dietary fiber, and trichostatin A (TSA) are known to downregulate NRP1 expression [60]. Butyrate diminishes the recruitment of Sp1 to the *NRP1* promoter in colorectal cancer, whereas the effect of TSA on NRP1 expression in endothelial cells is mediated by semaphorine III [61].

There is an established link between ACE2 deficiency, hyperinflammation, and SARS-CoV-2 infection [62]. ACE2 deficiency is associated with the clinical features, such as hypertension, diabetes, and cardiovascular disease that result in more severe COVID-19. The dysregulation between the AgII/AT1R and AT2R/Ang 1–7/MasR axes, favoring the latter, could give rise to an inflammatory state that could be blocked by the additional effect of VPA on the NF-kB pathway.

One of the best known effects of VPA is its ability to attenuate inflammation. In COVID-19 patients, the hyperproduction of proinflammatory cytokines, such as IL-1, IL-6, IL-12, IFN-γ, and TNF-α, helps determine the severity of the disease [63]. Accordingly, many experimental treatments have been proposed with the aim of limiting the extent of induction of inflammatory cytokines after SARS-CoV-2 infection. In our model, VPA reduces the expression of the pro-inflammatory cytokines TNF-α and IL-6 even after SARS-CoV-2 infection, making VPA a promising candidate for the treatment of COVID-19 patients. Similar effects were observed in monocytes and macrophages activated with lipopolysaccharide and in a variety of mouse models of inflammation in which VPA suppresses the expression of inflammatory cytokines in a NF-kB pathway-dependent manner [28, 48, 64]. VPA alters the acetylation of p65 by decreasing its DNA-binding affinity. Several studies have shown that acetylation and deacetylation of specific lysine residues in the p65 subunit regulate NF-kB-mediated gene expression [65–68]. Changes in the acetylation dynamics in each of the seven acetylated lysines modulate the DNA binding, the assembly with IkBα, the transcriptional activity of specific genes, and the length of the inflammatory response [69–71]. In this way, acetylation of K122 and K123 causes p65 to detach from the DNA and exit the cytoplasm, thereby ending the NF-kB-mediated inflammatory response. HDAC3 is involved in reversing these processes by deacetylating specific lysine residues and, consequently, the activation of the inflammatory response. In fact, blockage of HDAC3 activity by selective inhibitors produces an anti-inflammatory effect in inflammatory lung diseases and LPS-activated macrophages [72, 73]. VPA reverts the HDAC-3-mediated deacetylation of p65, attenuating the expression of specific inflammatory genes regulated by NF-kB [49, 74].

In addition to VPA’s role as an HDAC inhibitor, the drug has multiple effects and molecular targets, such as interfering with the metabolism of membrane lipids, which boosts expression of phosphatidylcholine pathway genes or reduces that of phosphatidylinositol [75, 76]. In this way, alterations in the host cell membrane could impair the assembly and production of some viruses. Vazquez-Calvo et al. [31] showed that the treatment of cells with VPA fully abolished the production of infectious envelope viruses but did not change the production of nonenveloped viruses. Surprisingly, the effect was maintained when VPA was added after infection. However, although VPA did not alter the number of budding viral particles, it produced particles that are more unstable in the infection medium. These results suggest that the antiviral potential of VPA could be mediated by the modifications in the cellular membrane of the host that use the envelope viruses to release new viral particles [32]. Furthermore, VPA diminishes the yields of infectious HSV-1 particles [77], and our results show that VPA not only decreases the viral infectivity of the enveloped SARS-CoV-2 virus by inhibiting ACE2 and NRP1 expression, but also reduces the generation of infectious SARS-CoV-2 particles without interfering with the synthesis of viral proteins. VPA also inhibits the production of particles of VSV, another envelope virus, corroborating its antiviral effect independently of ACE2. Recent studies have indicated the potential of additional epigenetic remodelers to block SARS-CoV-2 production. Resveratrol, whose multiple biological properties include being an activator of SIRT1, reduces the viral titers by 80% in in vitro cultures, although the underlying mechanisms are unclear [78, 79]. Taken together, the evidence demonstrates that epigenetic mechanisms can regulate all the key viral processes: entry, replication and transcription, and egress of new and stable viral particles. Therefore, these mechanisms, some of which we do not yet fully understand, are potentially useful in conjunction with clinically approved epigenetic drugs in the fight against SARS-CoV-2.

In summary, our study demonstrates that VPA, a drug commonly used in patients with epilepsy and bipolar disorders, can influence the infectivity of SARS-CoV-2 and can dampen the production of inflammatory cytokines. However, we cannot ignore the fact that all the studies have been done using cell models and that although we used cell lines of different origins (tumoral, non-tumoral, and primary cells), the susceptibility and tolerance to diverse doses of VPA could vary. Therefore, further studies in preclinical models are needed to determine the minimal effective dose of VPA that not only inhibits the expression of SARS-CoV-2 receptors and inflammatory molecules, but also defines the therapeutic range over which it could be considered a potential antiviral drug. Additionally, large clinical studies evaluating the SARS-CoV-2 infection outcomes in VPA-treated patients for epilepsy treatment could help to determine the effectiveness within the therapeutic range currently used. An advantage is that VPA has not exhibited any adverse interactions with treatments currently used for COVID-19, such as hydroxychloroquine, lopanivir, and remdesivir [34], although clinical trials are needed to evaluate the safety and efficacy of such drug combinations.

## Conclusions

After more than one year’s experience of the SARS-CoV-2 pandemic, we know that a huge hyperinflammatory response is triggered in COVID-19 patients that contributes to a very poor disease prognosis, that the disease is more serious in patients with specific comorbidities (e.g., diabetes mellitus, obesity, and hypertension), and that there are currently no effective therapeutic treatments that limit the replication of the virus once infection has taken place. Here, we show that VPA interferes with two essential processes in the viral cycle: It can reduce the infectivity of SARS-CoV-2, through downregulation of its receptors ACE2 and NRP1, and decreases the viral yields, probably by modulating virus budding or by altering the composition of the envelope producing virions to greatly reduce its stability. We also corroborate the earlier finding that VPA blocks the expression of the NF-kB-mediated inflammatory cytokines in the context of infection mediated by SARS-CoV-2. Thus, until everyone in the world is vaccinated, we will have to evaluate the effectiveness of new and current treatments of COVID-19 disease. In this context, epigenetic compounds such as VPA, which shows antiviral and immunomodulatory effects, represent promising candidates for use as COVID-19 treatments.

## Methods

### Cell lines, viruses, and reagents

Several cell lines were used. The African green monkey kidney-derived epithelial Vero E6 cells and the human hepatocellular carcinoma cell line, Huh-7, were kindly provided by Luis Enjuanes (Centro Nacional de Biotecnología-CSIC). The HK-2 human proximal tubular cell line (CRL-3216™), human lung epithelial BEAS-2B cells (CRL-9609™), Caco-2 human colorectal adenocarcinoma cell line (HTB-37), and primary human umbilical vein endothelial cells (HUVECs) (CRL-1730™) were all obtained from the American Type Culture collection. Huh-7, Vero E6, and Caco-2 cells were grown in Dulbecco's modified Eagle's medium (DMEM) (Gibco, Invitrogen, CA) supplemented with 25 mM HEPES and 10% fetal bovine serum (Fisher). HK-2 cell line was grown in RPMI-1640 medium (Gibco) supplemented with 10% fetal bovine serum (Fisher), 1% Insulin-transferrin-selenium (Gibco) and 36 mg/ml of hydrocortisone (Sigma-Aldrich). BEAS-2B was maintained in LHC-9 serum-free medium (Gibco) and HUVECs cultured in a mixture of DMEM and Ham’s-F12 medium (1:1) supplemented with 10% fetal bovine serum (Fisher), 50 µg/ml ECGS (Sigma) and 0.1 mg/ml heparin (Sigma). All media were further supplemented with 100 U/ml penicillin and 100 μg/ml streptomycin (Gibco).

SARS-CoV-2, isolated in Vero E6 cells, originating from a nasal swab from a patient infected in Madrid, Spain (unpublished results), and human coronavirus-229E (HCoV-229E) were kindly provided by Luis Enjuanes (Centro Nacional de Biotecnología-CSIC). Vesicular stomatitis virus expressing green fluorescent protein (VSV-GFP) has been previously described [80].

Valproic acid sodium salt (VPA; Sigma) was dissolved directly in culture medium at the indicated concentrations. The HDAC inhibitors Trichostatin A (TSA), PCI-24781 (specific for class I and IIb HDACs), TMP-195 (specific for class IIa HDACs), and sirtinol (specific for SIRT1 and 2, class III HDACs), obtained from Selleckchem, were prepared in DMSO.

### Virus infections

Confluent monolayers of Huh-7 and HK-2 cells (24-well-format plates) were treated for 24 h with VPA at 4, 8, and 16 mM concentrations to enable the analysis of the prophylactic effect of VPA. Cells were then infected with SARS-CoV-2 (multiplicity of infection, MOI, 0.5), VSV-GFP (MOI 0.01), and HCoV-229E (MOI 0.1) for 24 and 48 h in the absence of VPA. Cell culture media were collected at 24 and 48 h post-infection (hpi) and titrated in Vero E6 cells as described below.

Confluent monolayers of Huh-7 and HK-2 cells (24-well-format plates) were infected with SARS-CoV-2 (MOI, 0.5), VSV-GFP (MOI 0.01), and HCoV-229E (MOI 0.1) to analyze the therapeutic effect of VPA. VPA was added to the media at 4, 8, and 16 mM concentrations 1 hpi. Media were collected and titrated 24 hpi. Additionally, at 6 and 16 hpi, SARS-CoV-2-infected cells (MOI, 0.5) were collected and used for total RNA purification, and others were collected in RIPA buffer (Cell Signaling Technology) supplemented with a protease inhibitor cocktail (Merck Millipore, MA, USA), and the cell extracts used for western blot analysis.

### Virus titrations

Vesicular stomatitis virus expressing green fluorescent protein (VSV-GFP) was titrated by plaque assay (plaque-forming units per milliliter, PFU/ml) in Vero E6 cells. Confluent cell monolayers (24-well format) were infected with tenfold serial dilutions for 1 h at 37 °C, overlaid with 0.7% agar, and incubated at 37 °C for 1 day. HCoV-229E was titrated by plaque assay in Huh-7 cells. Confluent cell monolayers (24-well format) were infected with tenfold serial dilutions for 1 h at 33 °C, overlaid with 0.7% agar, and incubated at 33 °C for 4 days. SARS-CoV-2 virus titrations were performed in Vero E6 cells grown in 24-well plates and infected with tenfold serial dilutions of the virus. After 1-h absorption, cells were overlaid with low electroendosmosis agarose (Pronadisa) and incubated for 3 days at 37 °C. For all virus titrations, cells were fixed with 10% formaldehyde in phosphate buffer saline (PBS) and permeabilized with 20% methanol. Viral plaques were visualized and counted using crystal violet.

### Western blot

Total protein samples from untreated and VPA-treated cultured cells were isolated in RIPA buffer (Cell Signaling Technology) supplemented with a protease inhibitor cocktail (Merck Millipore, MA, USA) for 30 min on ice. Proteins were quantified using Bradford protein assay (Bio-Rad) and 20–50 µg per lane were separated on 10% polyacrylamide-SDS gels, blotted onto 0.45-μm Immobilon-E PVDF membranes (Merck Millipore, MA, USA), and detected by western blot analysis. The following primary antibodies were used: ACE-2 antibody-AC18F (NBP2-80035, Novus Biologicals, CO, USA), recombinant anti-NRP1 antibody [ERP3113] (ab81321, Abcam, UK), SARS-CoV-2-nucleocapsid protein antibody (GTX135357, GeneTex, CA, USA), and β-actin antibody (13E5; Cell Signaling, MA, USA). Secondary donkey anti-rabbit (406401, Biolegend) and goat anti-mouse (10799354, Invitrogen) antibodies conjugated with HRP were used. Immunoreactive bands were visualized using Immobilon Forte Western HRP substrate (Merck Millipore, MA, USA) and quantified with ImageJ version 1.53c software (NIH).

### Gene expression studies

Total RNA from untreated or VPA- and TNF-α-treated cell lines was isolated using a GeneMATRIX Universal RNA purification kit (EURx, Poland) following the manufacturer’s instructions. For virus infections, RNAs from mock-infected or infected cells were extracted using a total RNA extraction kit (Omega Bio-tek, GA, USA) following the manufacturer's recommendations. Purified RNA (1 µg) was reverse-transcribed to cDNA using a high-capacity cDNA reverse-transcription kit (Applied Biosystems). Quantification was performed by reverse transcription–polymerase chain reaction (RT-PCR) using a TaqMan gene expression assay (Applied Biosystems) for the human *TNFA* (Hs00174128_m1), *IL6* (Hs00174131_m1), *ACE2* (Hs01085331_m1), and *GAPDH* (Hs02786624_g1) genes with TaqMan gene expression master mix (Applied Biosystems). Expression of the *NRP1, CTSL1, DPP4*, and *RFX5* genes was analyzed using TB Green Premix Ex Taq (Takara, Japan) and the following primers: *NRP1*: 5′-TTCAGGATCACACAGGAGATGG-3′ (sense) and 5′-TAAACCACAGGGCTCACCAG-3′ (antisense); *CTSL1*: 5′-AGGCATTTATTTTGAGCCAG-3′ (sense) and 5′-AATTCCACAATGGTTTCTCC-3′ (antisense); *DPP4*: 5′-GAAGAGAGGATTCCAAACAAC-3′ (sense) and 5′-CATTGTTCCAAACATATGCC-3′ (antisense) and RFX5: 5′-CTGATGCTAAGAGCCCCAAC-3′ (sense) and 5′-TCAGTGTGCTCTTCCAGGTG-3′ (antisense). To analyze the expression of the nucleocapsid phosphoprotein (*N*) gene from SARS-CoV-2, the 5′-GCCTCTTCTCGTTCCTCATCAC-3′ (sense) and 5′-AGCAGCATCACCGCCATTG-3′ (antisense) primers were used with the Power SYBR Green master mix (Thermo Fisher Scientific). In both cases, for NRP1 and N genes, the GAPDH gene was used to normalize the data using the 5′-TGCCATGGGTGGAATCATATTGGA-3′ (sense) and 5′-TCGGAGTCAACGGATTTGGGTCGT-3′ (antisense) primers.

### Apoptosis and proliferation assays

To analyze cellular viability, apoptotic and dead cells were quantified using the FITC Annexin V apoptosis detection kit and 7-AAD (Biolegend), following the manufacturer’s instructions. BEAS-2B, HK-2, Huh-7, Caco-2, and HUVEC cells were plated in 6-well plates at a seeding density of 3 × 10^6^ cells/well and a range of concentrations of VPA (1, 2, 4, 8, and 16 mM) were added for 24 h. After that, cells were removed and stained using the FITC-Annexin V Apoptosis detection kit with 7AAD (Biolegend), following the manufacturer’s instructions, and analyzed on a Gallios Flow Cytometer (Beckman Coulter, Inc.). Early apoptotic cells were considered as Annexin V + 7AAD- and late apoptotic/necrotic cells as Annexin V + 7AAD + .

The effect of VPA on cell proliferation was analyzed with an MTT (3-(4,5-dimethylthiazol-2-yl)-2,5-diphenyltetrazolium bromide) assay. Huh-7 and HK-2 cells were seeded at a density of 5 × 10^3^ cells/well in 96-well plates at the same VPA concentrations, as described above, for 24 h. Depending on the experimental requirements, medium was then replaced, and cells were cultured for a further 24 or 48 h or kept unreplaced for direct analysis. During the final 4 h, 0.5 mg/ml of MTT substrate was added to the cell culture. After that, the medium was removed, and formazan crystals were dissolved with DMSO. Absorbance at 570 nm was read by a spectrophotometer (Bio-Rad).

### Data analysis

VPA-treated and VPA-untreated cells were compared by Student’s unpaired-samples t test and the Mann–Whitney U test using SPSS Statistics for Windows, Version 20.0 (IBM Corp., Armonk, NY, USA). Data are represented as the mean and standard deviation (SD) of at least three independent experiments. Differences were considered statistically significant for values of p < 0.05.

## Supplementary Information

**Additional file 1: Fig. S1**. Effect of the treatment with VPA on cell viability of different cell lines. All cell lines (BEAS-2B, HK-2, Huh-7, CACO-2, and HUVEC) were treated with VPA (1, 2, 4, 8, and 16 mM) for 24. Control cells were untreated and only grown with culture medium. After that, cells were stained with annexin V-FITC and 7AAD and early apoptotic (annexin V-FITC + and 7AAD−) and late apoptotic / necrotic (annexin V-FITC + and 7AAD +) cells were quantified by flow cytometry. (A) Data are represented as the percentage of necrotic and apoptotic cells at 24 h post-treatment with VPA. (B) Representative dot-plots of Annexin V/7AAD for each cell line after 24 h of VPA treatment. Numbers in the lower and upper right quadrants show the percentage of early apoptotic and late apoptotic/necrotic cells, respectively.

**Additional file 2: Fig. S2**. Expression of the *CTSL1*, *DPP4*, and *RFX5* genes after VPA treatment in HK-2 and Huh-7 cell lines. HK-2 and Huh-7 cells were treated with different doses of VPA (1, 2, 4, and 8 mM) for 24 h or untreated (control). Expression of *CTSL1*, *DPP4* and *RFX5* genes was assayed by RT-qPCR and *GADPH* was used as endogenous controls to quantify mRNA levels. Transcription levels were calculated by the 2^−ΔCT^ method (ΔCT: CT gene test—CT endogenous control). Data are presented as the mean ± SD of two independent experiments.

**Additional file 3:** **Fig. S3**. Treatment with HDAC inhibitors reduces the transcriptional levels of ACE-2 and NRP1. HK-2 and Huh-7 cell lines were treated with different histone deacetylase (HDAC) inhibitors; trichostatin A (TSA, pan-HDAC, 200–400 nM), PCI-24781 (specific for class I and IIb HDACs, 1 and 2 µM), TMP-195 (specific for class IIa HDACs, 5 and 10 µM) and sirtinol (specific for SIRT1 and SIRT2, 5 and 10 µM) were used. All inhibitors were prepared in DMSO, which was also used as the control. Expression of the ACE2 and NRP1 receptors was assayed by RT-qPCR using *GADPH* gene as endogenous controls. Transcription levels were calculated by the 2^−ΔCT^ method (ΔCT: CT gene test—CT endogenous control). Data are presented as the mean ± SD of at least three independent experiments. * p < 0.05.

**Additional file 4: Fig. S4**. Effect of VPA pretreatment on cell proliferation of HK-2 and Huh-7 cell lines. HK-2 and Huh-7 cell lines were treated with VPA (1, 2, 4, 8, and 16 mM) for 24 h. At each particular time, culture medium was replaced by fresh medium without VPA, and cells were grown for an additional 24 and 48 h. Control cells were untreated and only grown with culture medium. Cell proliferation was quantified by fluorometric MTT assay and data are shown as the percentage of cells compared with that of control cells, represented as the mean ± SD of triplicate measures.

**Additional file 5: Fig. S5**. Effect of VPA pretreatment on SARS-CoV-2 infectivity. HK-2 and Huh-7 cells were treated during 24 h with VPA at 4, 8, and 16 mM, or left untreated (control cells). After 24 h, culture medium was replaced by fresh medium without VPA and cells were infected with SARS-CoV-2, VSV-GFP, or HCoV-229E, and virus titers were determined by plaque assay at 24 and 48 hpi, as in Fig. 3, and represented as the percentage of the titers compared with the titers measured in control cells. Data are represented as the mean ± SD of absolute frequencies from triplicate measures. * p < 0.05 compared with control.

**Additional file 6: Fig. S6**. Therapeutic effect of VPA on viral yield after SARS-CoV-2 infection. HK-2 and Huh-7 cells were infected with SARS-CoV-2 (**A**) or with VSV-GFP (**B**), and 1 h after infection, cells were treated VPA at 4, 8, and 16 mM, or left untreated (control cells). Virus titers were determined by plaque assay at 24 hpi, as in Fig. 5, and represented as the percentage of the titers compared with the titers measured in control cells. Data are represented as the mean ± SD of absolute frequencies from triplicate measures. * p < 0.05.

**Additional file 7: Fig. S7**. Effect of VPA on cell proliferation of HK-2 and Huh-7 cell lines. HK-2 and Huh-7 cell lines were treated with VPA (1, 2, 4, 8, and 16 mM) for 24 h. Control cells were untreated and only grown with culture medium. Cell proliferation was quantified by fluorometric MTT assay and data are shown as the percentage of cells compared with control cells, represented as the mean ± SD from triplicate measures.

## Data Availability

All data generated or analyzed during this study are included in this published article and its supplementary information files.

## Abbreviations

ACE2: Angiotensin-converting enzyme 2
AngII: Angiotensin II
ARDS: Acute respiratory syndrome
AT1R: Angiotensin II receptor type 1
COVID-19: Coronavirus-induced disease 2019
gRNA: Genomic messenger RNA
HCoV-229E: Human coronavirus 229E
HDAC: Histone deacetylase enzyme
hpi: Hours post-infection
HSV-1: Herpes simplex virus-1
MOI: Multiplicity of infection
NELF: Negative elongation factor
NRP1: Neuropilin-1
PFU: Plaque-forming units
sg mRNA: Subgenomic messenger RNA
TSA: Trichostatin A
VPA: Valproic acid
VSV: Vesicular stomatitis virus
