# Supplementary material for: Epigenetic targeting of the ACE2 and NRP1 viral receptors limits SARS-CoV-2 infectivity
Source: Clin Epigenetics. 2021 Oct 11;13:187. doi: 10.1186/s13148-021-01168-5 (PMC8504098; doi:10.1186/s13148-021-01168-5)
Supplement: Supplementary file 1 — Additional file 1: Fig. S1. Effect of the treatment with VPA on cell viability of different cell lines. All cell lines (BEAS-2B, HK-2, Huh-7, CACO-2, and HUVEC) were treated with VPA (1, 2, 4, 8, and 16 mM) for 24. Control cells were untreated and only grown with culture medium. After that, cells were stained with annexin V-FITC and 7AAD and early apoptotic (annexin V-FITC + and 7AAD−) and late apoptotic / necrotic (annexin V-FITC + and 7AAD +) cells were quantified by flow cytometry. (A) Data are represented as the percentage of necrotic and apoptotic cells at 24 h post-treatment with VPA. (B) Representative dot-plots of Annexin V/7AAD for each cell line after 24 h of VPA treatment. Numbers in the lower and upper right quadrants show the percentage of early apoptotic and late apoptotic/necrotic cells, respectively. [file 13148_2021_1168_MOESM1_ESM.pptx]

## Slide 1
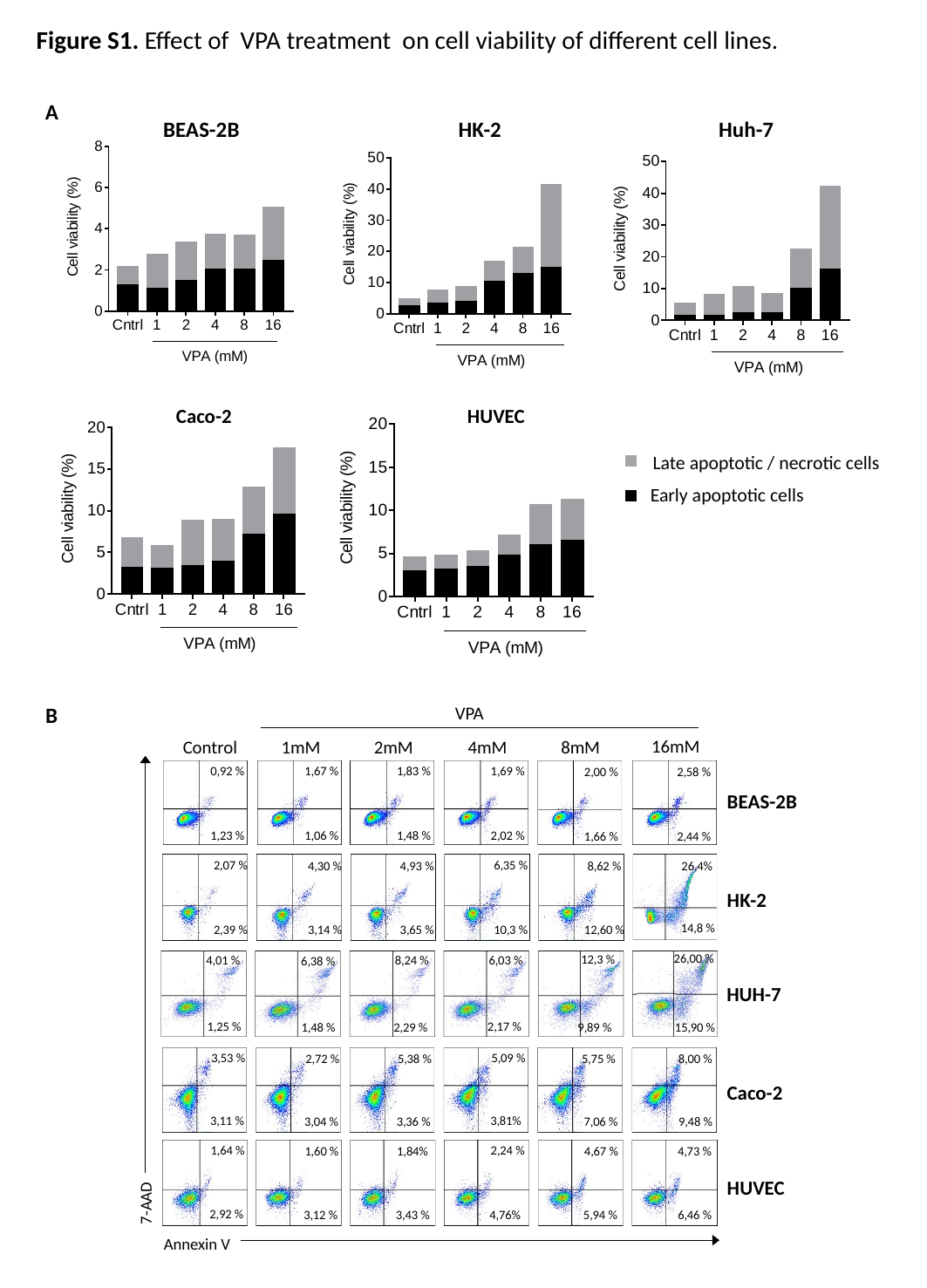

Figure S1. Effect of VPA treatment on cell viability of different cell lines.
A
BEAS-2B
HK-2
Huh-7
Caco-2
HUVEC
Late apoptotic / necrotic cells
Early apoptotic cells
B
VPA
16mM
Control
1mM
2mM
4mM
8mM
0,92 %
1,69 %
1,67 %
1,83 %
2,00 %
2,58 %
BEAS-2B
1,23 %
2,02 %
1,06 %
1,48 %
1,66 %
2,44 %
2,07 %
6,35 %
4,30 %
4,93 %
26,4%
8,62 %
HK-2
14,8 %
2,39 %
10,3 %
3,14 %
3,65 %
12,60 %
26,00 %
12,3 %
6,03 %
4,01 %
8,24 %
6,38 %
HUH-7
1,25 %
2,17 %
1,48 %
2,29 %
15,90 %
9,89 %
3,53 %
5,09 %
2,72 %
5,38 %
5,75 %
8,00 %
Caco-2
3,11 %
3,81%
3,04 %
3,36 %
7,06 %
9,48 %
1,64 %
2,24 %
1,60 %
1,84%
4,67 %
4,73 %
HUVEC
7-AAD
2,92 %
4,76%
3,12 %
3,43 %
5,94 %
6,46 %
Annexin V
