# Supplementary material for: Epigenetic targeting of the ACE2 and NRP1 viral receptors limits SARS-CoV-2 infectivity
Source: Clin Epigenetics. 2021 Oct 11;13:187. doi: 10.1186/s13148-021-01168-5 (PMC8504098; doi:10.1186/s13148-021-01168-5)
Supplement: Supplementary file 2 — Additional file 2: Fig. S2. Expression of the CTSL1, DPP4, and RFX5 genes after VPA treatment in HK-2 and Huh-7 cell lines. HK-2 and Huh-7 cells were treated with different doses of VPA (1, 2, 4, and 8 mM) for 24 h or untreated (control). Expression of CTSL1, DPP4 and RFX5 genes was assayed by RT-qPCR and GADPH was used as endogenous controls to quantify mRNA levels. Transcription levels were calculated by the 2−ΔCT method (ΔCT: CT gene test—CT endogenous control). Data are presented as the mean ± SD of two independent experiments. [file 13148_2021_1168_MOESM2_ESM.pptx]

## Slide 1
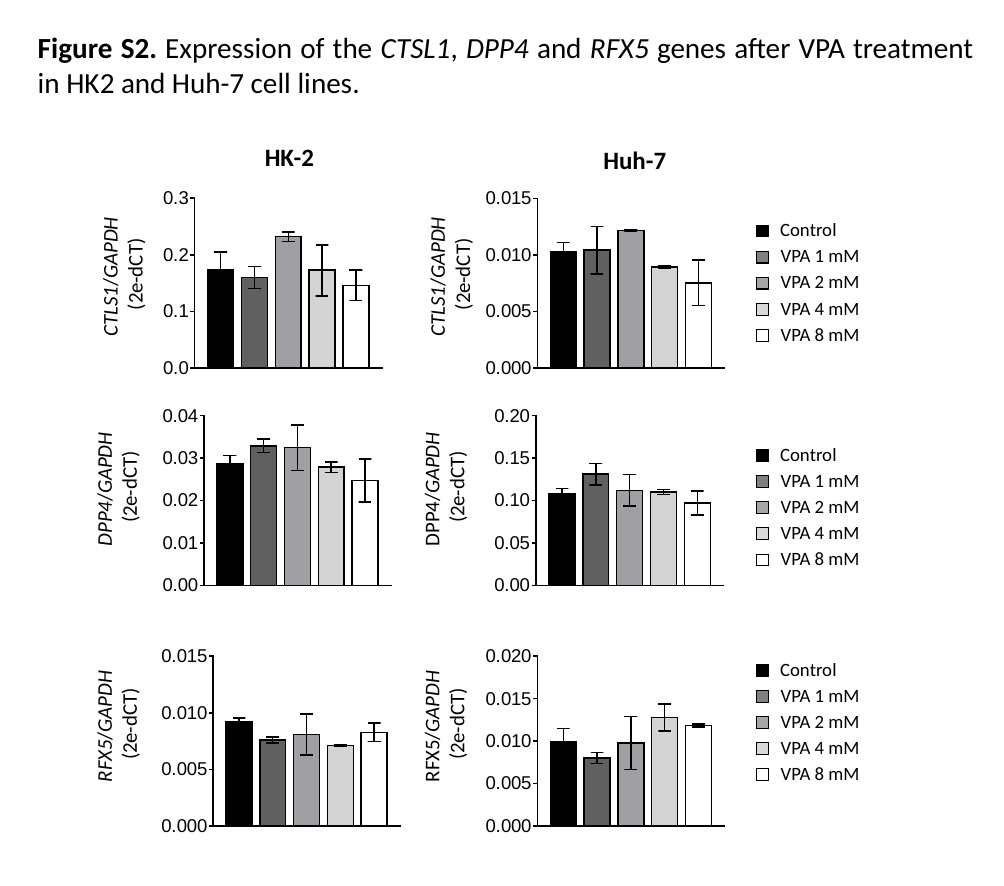

Figure S2. Expression of the CTSL1, DPP4 and RFX5 genes after VPA treatment in HK2 and Huh-7 cell lines.
HK-2
Huh-7
Control
VPA 1 mM
VPA 2 mM
VPA 4 mM
VPA 8 mM
CTLS1/GAPDH
(2e-dCT)
CTLS1/GAPDH
(2e-dCT)
Control
VPA 1 mM
VPA 2 mM
VPA 4 mM
VPA 8 mM
DPP4/GAPDH
(2e-dCT)
DPP4/GAPDH
(2e-dCT)
Control
VPA 1 mM
VPA 2 mM
VPA 4 mM
VPA 8 mM
RFX5/GAPDH
(2e-dCT)
RFX5/GAPDH
(2e-dCT)
