# Supplementary material for: Epigenetic targeting of the ACE2 and NRP1 viral receptors limits SARS-CoV-2 infectivity
Source: Clin Epigenetics. 2021 Oct 11;13:187. doi: 10.1186/s13148-021-01168-5 (PMC8504098; doi:10.1186/s13148-021-01168-5)
Supplement: Supplementary file 3 — Additional file 3: Fig. S3. Treatment with HDAC inhibitors reduces the transcriptional levels of ACE-2 and NRP1. HK-2 and Huh-7 cell lines were treated with different histone deacetylase (HDAC) inhibitors; trichostatin A (TSA, pan-HDAC, 200–400 nM), PCI-24781 (specific for class I and IIb HDACs, 1 and 2 µM), TMP-195 (specific for class IIa HDACs, 5 and 10 µM) and sirtinol (specific for SIRT1 and SIRT2, 5 and 10 µM) were used. All inhibitors were prepared in DMSO, which was also used as the control. Expression of the ACE2 and NRP1 receptors was assayed by RT-qPCR using GADPH gene as endogenous controls. Transcription levels were calculated by the 2−ΔCT method (ΔCT: CT gene test—CT endogenous control). Data are presented as the mean ± SD of at least three independent experiments. * p < 0.05. [file 13148_2021_1168_MOESM3_ESM.pptx]

## Slide 1
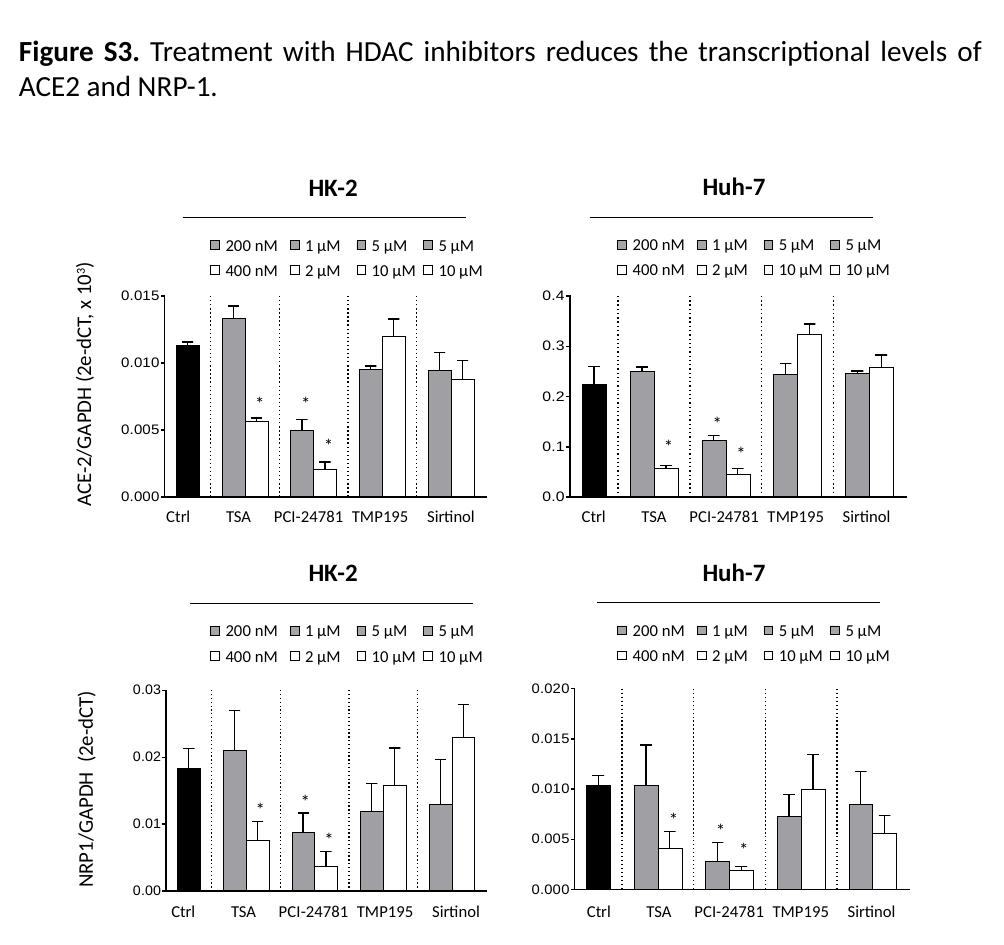

Figure S3. Treatment with HDAC inhibitors reduces the transcriptional levels of ACE2 and NRP-1.
Huh-7
HK-2
200 nM
1 µM
5 µM
5 µM
400 nM
2 µM
10 µM
10 µM
200 nM
1 µM
5 µM
5 µM
400 nM
2 µM
10 µM
10 µM
ACE-2/GAPDH (2e-dCT, x 103)
*
*
*
*
*
*
Ctrl
TSA
PCI-24781
TMP195
Sirtinol
Ctrl
TSA
PCI-24781
TMP195
Sirtinol
Huh-7
HK-2
200 nM
1 µM
5 µM
5 µM
400 nM
2 µM
10 µM
10 µM
200 nM
1 µM
5 µM
5 µM
400 nM
2 µM
10 µM
10 µM
NRP1/GAPDH (2e-dCT)
*
*
*
*
*
*
Ctrl
TSA
PCI-24781
TMP195
Sirtinol
Ctrl
TSA
PCI-24781
TMP195
Sirtinol
