# Supplementary material for: Epigenetic targeting of the ACE2 and NRP1 viral receptors limits SARS-CoV-2 infectivity
Source: Clin Epigenetics. 2021 Oct 11;13:187. doi: 10.1186/s13148-021-01168-5 (PMC8504098; doi:10.1186/s13148-021-01168-5)
Supplement: Supplementary file 5 — Additional file 5: Fig. S5. Effect of VPA pretreatment on SARS-CoV-2 infectivity. HK-2 and Huh-7 cells were treated during 24 h with VPA at 4, 8, and 16 mM, or left untreated (control cells). After 24 h, culture medium was replaced by fresh medium without VPA and cells were infected with SARS-CoV-2, VSV-GFP, or HCoV-229E, and virus titers were determined by plaque assay at 24 and 48 hpi, as in Fig. 3, and represented as the percentage of the titers compared with the titers measured in control cells. Data are represented as the mean ± SD of absolute frequencies from triplicate measures. * p < 0.05 compared with control. [file 13148_2021_1168_MOESM5_ESM.pptx]

## Slide 1
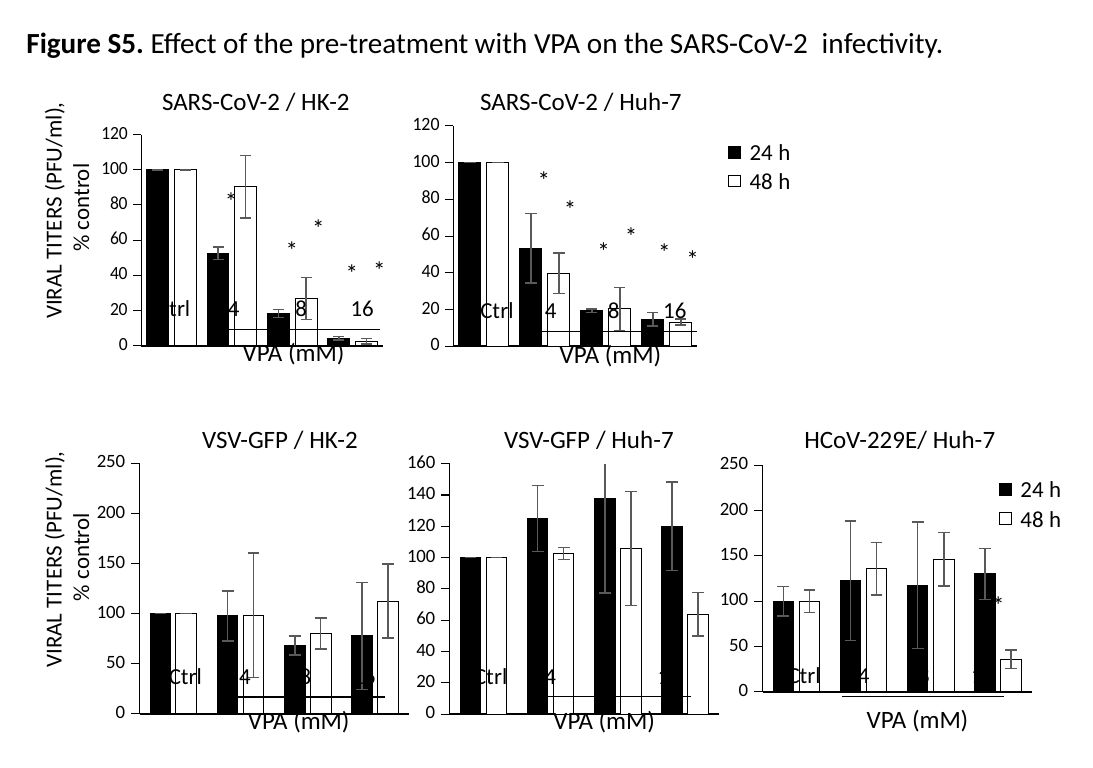

Figure S5. Effect of the pre-treatment with VPA on the SARS-CoV-2 infectivity.
SARS-CoV-2 / HK-2
SARS-CoV-2 / Huh-7
### Chart
| Category | | |
|---|---|---|
| control | 100.0 | 100.0 |
| 4mM | 52.5 | 90.35947712418297 |
| 8mM | 18.33333333333333 | 26.90631808278867 |
| 16mM | 4.333333333333333 | 2.652505446623094 |
### Chart
| Category | | |
|---|---|---|
| control | 100.0 | 100.0 |
| 4mM | 53.33333333333334 | 39.80952380952381 |
| 8mM | 19.33333333333333 | 20.28571428571428 |
| 16mM | 14.66666666666667 | 13.14285714285714 |24 h
48 h
*
VIRAL TITERS (PFU/ml),
 % control
*
*
*
*
*
*
*
*
*
*
Ctrl
4
8
16
Ctrl
4
8
16
VPA (mM)
VPA (mM)
VSV-GFP / HK-2
VSV-GFP / Huh-7
HCoV-229E/ Huh-7
### Chart
| Category | | |
|---|---|---|
| control | 100.0 | 100.0 |
| 4mM | 97.69230769230768 | 98.42657342657344 |
| 8mM | 68.26923076923077 | 80.06993006993007 |
| 16mM | 77.69230769230768 | 112.4125874125874 |
### Chart
| Category | | |
|---|---|---|
| control | 100.0 | 100.0 |
| 4mM | 125.0 | 102.6315789473684 |
| 8mM | 137.5 | 105.7894736842105 |
| 16mM | 120.0 | 63.8421052631579 |
### Chart
| Category | | |
|---|---|---|
| control | 100.0 | 100.0 |
| 4 mM | 122.5 | 135.7142857142857 |
| 8 mM | 117.5 | 146.4285714285714 |
| 16 mM | 130.0 | 35.71428571428572 |24 h
48 h
VIRAL TITERS (PFU/ml),
 % control
*
Ctrl
4
8
16
Ctrl
4
8
16
Ctrl
4
8
16
VPA (mM)
VPA (mM)
VPA (mM)
