# Supplementary material for: Epigenetic targeting of the ACE2 and NRP1 viral receptors limits SARS-CoV-2 infectivity
Source: Clin Epigenetics. 2021 Oct 11;13:187. doi: 10.1186/s13148-021-01168-5 (PMC8504098; doi:10.1186/s13148-021-01168-5)
Supplement: Supplementary file 6 — Additional file 6: Fig. S6. Therapeutic effect of VPA on viral yield after SARS-CoV-2 infection. HK-2 and Huh-7 cells were infected with SARS-CoV-2 (A) or with VSV-GFP (B), and 1 h after infection, cells were treated VPA at 4, 8, and 16 mM, or left untreated (control cells). Virus titers were determined by plaque assay at 24 hpi, as in Fig. 5, and represented as the percentage of the titers compared with the titers measured in control cells. Data are represented as the mean ± SD of absolute frequencies from triplicate measures. * p < 0.05. [file 13148_2021_1168_MOESM6_ESM.pptx]

## Slide 1
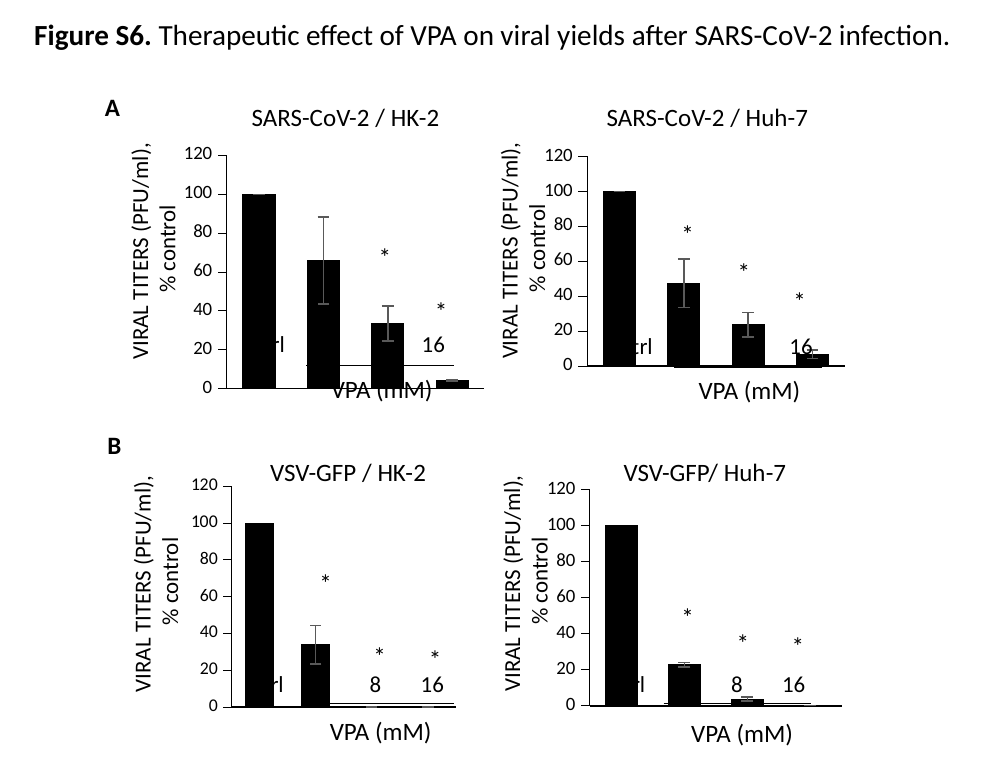

Figure S6. Therapeutic effect of VPA on viral yields after SARS-CoV-2 infection.
A
SARS-CoV-2 / HK-2
SARS-CoV-2 / Huh-7
### Chart
| Category | |
|---|---|
| control | 100.0 |
| 4 mM | 47.32142857142856 |
| 8 mM | 23.66071428571428 |
| 16 mM | 6.785714285714286 |*
*
*
Ctrl
4
8
16
VPA (mM)
### Chart
| Category | |
|---|---|
| control | 100.0 |
| 4mM | 65.9090909090909 |
| 8mM | 33.63636363636364 |
| 16mM | 4.272727272727272 |VIRAL TITERS (PFU/ml),
 % control
VIRAL TITERS (PFU/ml),
 % control
*
*
Ctrl
4
8
16
VPA (mM)
B
VSV-GFP / HK-2
VSV-GFP/ Huh-7
### Chart
| Category | |
|---|---|
| control | 100.0 |
| 4 mM | 22.72727272727273 |
| 8 mM | 3.636363636363637 |
| 16 mM | 0.000386363636363636 |*
*
*
Ctrl
4
8
16
VPA (mM)
### Chart
| Category | |
|---|---|
| control | 100.0 |
| 4 mM | 33.8235294117647 |
| 8 mM | 0.0107352941176471 |
| 16 mM | 2.94117647058824e-05 |VIRAL TITERS (PFU/ml),
 % control
VIRAL TITERS (PFU/ml),
 % control
*
*
*
Ctrl
4
8
16
VPA (mM)
