# Supplementary material for: Epigenetic targeting of the ACE2 and NRP1 viral receptors limits SARS-CoV-2 infectivity
Source: Clin Epigenetics. 2021 Oct 11;13:187. doi: 10.1186/s13148-021-01168-5 (PMC8504098; doi:10.1186/s13148-021-01168-5)
Supplement: Supplementary file 7 — Additional file 7: Fig. S7. Effect of VPA on cell proliferation of HK-2 and Huh-7 cell lines. HK-2 and Huh-7 cell lines were treated with VPA (1, 2, 4, 8, and 16 mM) for 24 h. Control cells were untreated and only grown with culture medium. Cell proliferation was quantified by fluorometric MTT assay and data are shown as the percentage of cells compared with control cells, represented as the mean ± SD from triplicate measures. [file 13148_2021_1168_MOESM7_ESM.pptx]

## Slide 1
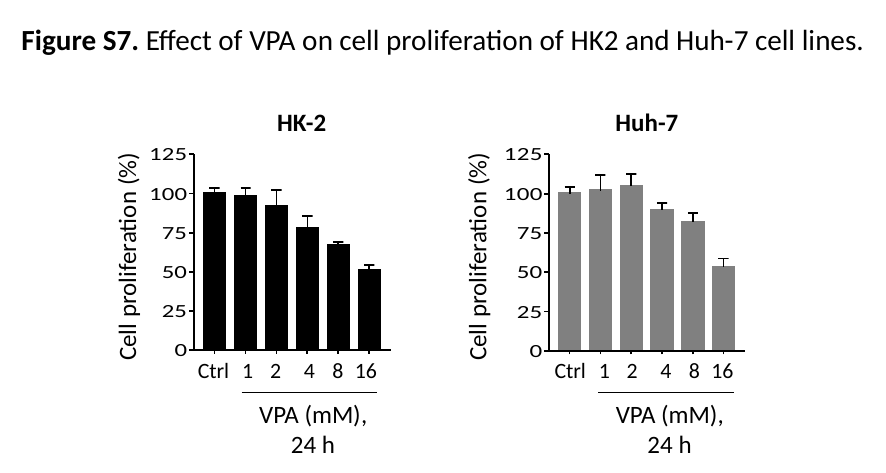

Figure S7. Effect of VPA on cell proliferation of HK2 and Huh-7 cell lines.
HK-2
Huh-7
Ctrl
1
2
4
8
16
VPA (mM),
24 h
Ctrl
1
2
4
8
16
VPA (mM),
24 h
Cell proliferation (%)
Cell proliferation (%)
